# Greenhouse covering cultivation promotes chlorophyll accumulation of tea plant (*Camellia sinensis*) by activating relevant gene expression and enzyme activity

**DOI:** 10.1186/s12870-024-05149-7

**Published:** 2024-05-24

**Authors:** Xueming Ma, Jixian Liu, Haiyan Li, Wenzhuo Wang, Lei Liu, Peiqiang Wang, Jianhui Hu, Xinfu Zhang, Fengfeng Qu

**Affiliations:** 1 College of Horticulture, Qingdao Agricultural University, Qingdao, 266109 China; 2 Bureau of Agriculture and Rural Affairs of Laoshan District, Qingdao, 266061 China

**Keywords:** Plastic greenhouse covering, Tea plants, Chlorophyll metabolism, Gene expression

## Abstract

**Background:**

The tea plant (*Camellia sinensis* (L.) O. Kuntze) is one of the most economically important woody crops. Plastic greenhouse covering cultivation has been widely used in tea areas of northern China. Chlorophyll is not only the crucial pigment for green tea, but also plays an important role in the growth and development of tea plants. Currently, little is known about the effect of plastic greenhouse covering cultivation on chlorophyll in tea leaves.

**Results:**

To investigate the effect of plastic greenhouse covering cultivation on chlorophyll in tea leaves, color difference values, chlorophyll contents, gene expression, enzyme activities and photosynthetic parameters were analyzed in our study. Sensory evaluation showed the color of appearance, liquor and infused leaves of greenhouse tea was greener than field tea. Color difference analysis for tea liquor revealed that the value of ∆L, ∆b and b/a of greenhouse tea was significantly higher than field tea. Significant increase in chlorophyll content, intracellular CO_2_, stomatal conductance, transpiration rate, and net photosynthetic rate was observed in greenhouse tea leaves. The gene expression and activities of chlorophyll-metabolism-related enzymes in tea leaves were also activated by greenhouse covering.

**Conclusion:**

The higher contents of chlorophyll a, chlorophyll b and total chlorophyll in greenhouse tea samples were primarily due to higher gene expression and activities of chlorophyll-metabolism-related enzymes especially, chlorophyll a synthetase (chlG), pheophorbide a oxygenase (PAO) and chlorophyllide a oxygenase (CAO) in tea leaves covered by greenhouse. In general, our results revealed the molecular basis of chlorophyll metabolism in tea leaves caused by plastic greenhouse covering cultivation, which had great significance in production of greenhouse tea.

**Supplementary Information:**

The online version contains supplementary material available at 10.1186/s12870-024-05149-7.

## Introduction

Tea region of north Yangtze river is the highest latitude areas of China with climate characteristics of low temperature, rare rainfall, strong winds and other typical characteristics of temperate monsoon climate [1]. Therefore, the tea plants grown in northern China are vulnerable to suffer from winter freezing damage and spring frost injury [2, 3]. In order to prevent tea plants from climate risk, the plastic greenhouse is usually used as one of the key techniques of tea cultivation. Prevent studies showed that the plastic greenhouse cultivation could accelerate the growth of tea plants, advance picking time, increase tea yield, and thereby improve economic benefits [4, 5]. Compared to open field cultivation, the plastic greenhouse cultivation obviously promotes plant growth and breaks plant dormancy by increasing the air temperature, ground temperature and effective accumulated temperature [6, 7]. So far, the plastic greenhouse cultivation has been spread throughout the northern tea region of China.

**Fig. 1:**
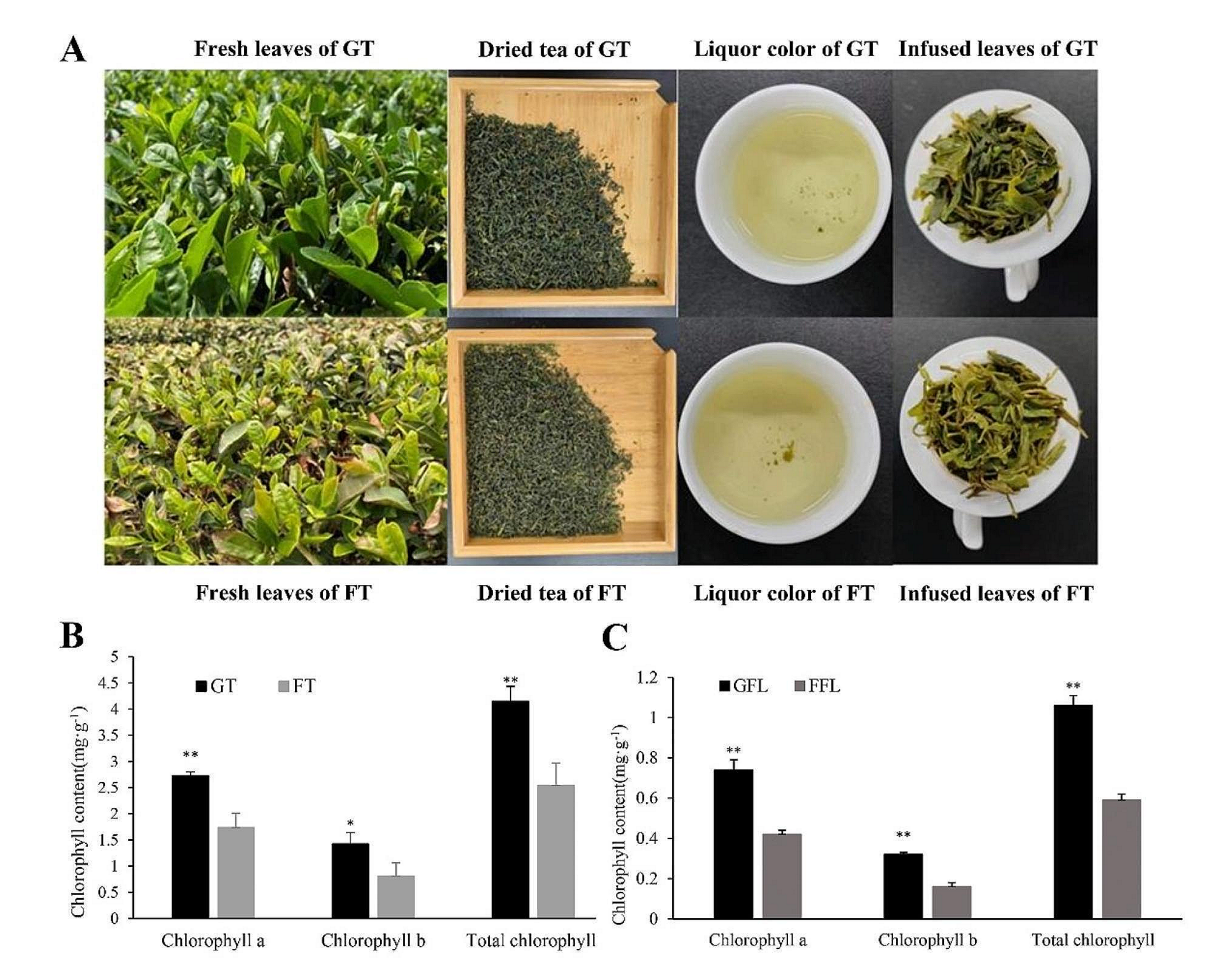
Color difference between greenhouse tea and field tea. **A**, the difference of sensory evaluation in GT and FT; **B**, the difference of chlorophyll content in GT and FT; C, the difference of chlorophyll content in GFL and FFL. Data are presented as mean ± standard deviation (*n* = 3); * indicates a significant difference at *P* < 0.05 level; ** indicates a significant difference at *P* < 0.01 level, GT, greenhouse tea; FT, field tea; GFL, greenhouse fresh leaves; FFL, field fresh leaves

Green tea has been consumed as a popular beverage worldwide because of its unique color, aroma and taste [8]. According to the national standard of China (GB/T 23776 − 2018), the green color of tea appearance, tea aroma and infused leaves is an important indicator of high-quality green tea [9]. And the green color of green tea is mainly owing to high chlorophyll contents [10]. The chlorophyll contents of tea leaves depend on multiple factors like tea cultivars, cultivation measures, processing methods [11–13]. Shading has been proved to be an effective measure to induce chlorophyll accumulation in tea leaves [14, 15]. However, shading can significantly decrease the leaf thickness, leaf internodes as well as photosynthetic rate, resulting in a reduction of tea yield [16]. Our preliminary investigation found that the fresh tea leaves grown in the plastic greenhouse were greener than those grown in the open field (Fig. 1A), which implied that the plastic greenhouse might increase the chlorophyll accumulation of tea leaves and avoid shading-induced tea yield losses. This study aimed to clarify the effect of plastic greenhouse on chlorophyll metabolism in tea leaves, which would provide scientific guidance for production of superior quality and high yield green tea.

## Materials and methods

### Chemicals and reagents

Absolute ethanol and acetone were purchased from Fuyu Fine Chemical Co., Ltd. (Tianjin, China). RNA isolation kit was purchased from Accurate Biotechnology (Hunan) Co., Ltd. (Changsha, China). RT reagent kit and SYBR Green Mix were purchased from Takara Biotechnology (Dalian) Co., Ltd. (Dalian, China). The ELISA kits of chlorophyll a synthetase (chlG), pheophorbide a oxygenase (PAO), magnesium dechelatase (SGR), chlorophyllase (CLH), chlorophyll(ide) b reductase (NOL/NYC1) and chlorophyllide a oxygenase (CAO) were purchased from Jiangsu Jingmei Biotechnology Co., Ltd. (Jiangsu, China).

### Plant materials and treatment

To protect tea plants from winter freezing damage, plastic-covered tunnel greenhouses were widely used in tea gardens of northern China. Usually, tea plants were covered by greenhouses in November and removed in April of next year. To save production cost, some tea plants overwintered naturally in the field. In April 2023, fresh one bud and two leaves were picked from *Camellia sinensis sv*. ‘Huangshanzhong’, which was grown in tea gardens of Laoshan, Qingdao, China (latitude 36°16’N, longitude 120°37’E, altitude 23 m). The temperature, relative humidity and carbon dioxide concentration of greenhouse was higher than field (Table S3). Fresh leaves collected from tea plants cultivated in the open field or inside greenhouse were evenly divided into two portions. One portion was immediately frozen in liquid nitrogen and stored in -80 °C. The other portion was processed to green tea [17]. The processing conditions were as follows: fresh tea leaves were spread indoors for 3–4 h and then fixated for 2 min at 180 ~ 200 °C. The enzyme-denatured leaves were dried at 110 °C until the moisture of tea was about 5%.

### Sensory evaluation

The quality of green tea was scored and described as previous studies [18, 19]. Samples were evaluated by five professional assessors. 3.0 g of samples were infused in white porcelain cups with 150 mL of freshly boiled water for 4 min. Then, the tea fusion was poured into a white porcelain bowl. The professional assessors were instructed to evaluate tea appearance, liquor, aroma, taste and infused leaves. The total sensory score was evaluated by quality scores using a 100 − point scale, in which 10% accounted for the appearance, 30% for the aroma, 15% for the liquor color, 35% for the taste and 10% for the infused leaves. Samples were assessed three times through blind evaluation.

### Color determination

The color change of dried tea and tea liquor was respectively measured by a colorimeter (CR-400, Konica Minolta, Japan). The index of L, a, b, ∆L, ∆a, ∆b, ∆E, b/a, C_ab_, S_ab_ and H_ab_ was used for describing the color difference [12].

### Photosynthetic parameter determination

The photosynthetic parameter was measured by a photosynthetic apparatus (CIRAS-3, Hansatech Instruments Ltd, United Kingdom). Firstly, the infrared instrument and the assimilation chamber were connected into an open gas path system to supply a stable carbon dioxide gas source to the assimilation chamber. Secondly, first leaf and second leaves were inserted into the assimilation chamber and appropriate light was given. After the carbon dioxide difference (CO_2_r) and water vapor difference (H_2_Or) were stabilized (± 0.5), then recorded the value of intracellular CO_2_ (Ci), stomatal conductance (Gs), transpiration rate (Tr), water use efficiency (WUE) and net photosynthetic rate (Pn).

### Chlorophyll determination

Determination of chlorophyll was performed based on the colorimetric method with minor modification [20]. 0.1 g of fresh tea leaves was mixed with 10 mL of extraction solvent (anhydrous ethanol: acetone: distilled water = 4.5: 4.5: 1) and the mixture was then placed in the dark until the leaves turned entirely white. The amounts of chlorophyll were calculated from the readings of absorption at 663 nm and 645 nm using microplate reader with multi-wavelength measurement system (HBS-1101, Droide, Shanghai, China). The contents were calculated using the following formulas:

Content of chlorophyll a (C_a_, mg∙L^− 1^) = 12.72A_663_ – 2.95A_645_,

Content of chlorophyll b (C_b_, mg∙L^− 1^) = 22.88A_645_ – 4.67A_663_,

Total chlorophyll content (C_T_, mg∙L^− 1^) = C_a_ + C_b_.

where A_663_ and A_645_ were the absorbance at 663 nm and 645 nm respectively.

Finally, the total chlorophyll contents were calculated as mg/g dried sample.

### Gene expression analysis

RNA isolation kit (AG21019, Accurate Biology, Changsha, China) and RT reagent kit (Takara, Dalian, China) were respectively used to isolate total RNA and synthesize the first-strand cDNA following the corresponding instruction of kits. The primers were designed using the Primer Premier 5.0 program (PREMIER Biosoft International, Palo Alto, CA, USA) (Table S1). The quantitative real-time polymerase chain reaction (qRT-PCR) was performed in a reaction mixture volume of 20 µL, containing 2 µL of template cDNA, 0.8 µL of forward primer, 0.8 µL of reverse primer (10 µM), 10 µL of SYBR Green Mix (Takara, Dalian, China) and 6.4 µL of deionized water. qRT-PCR was performed with minor modification [21]. The PCR conditions were as follows: 95 °C for 30 s and 40 cycles of 95 °C for 5 s and 60 °C for 30 s, followed by a melting curve analysis from 60 °C to 95 °C. Target mRNA levels were normalized to *CsGAPDH* levels. Data was calculated by 2^−ΔΔCT^ method [22].

### Enzyme activity analysis

The activities of chlorophyll a synthetase (chlG), pheophorbide a oxygenase (PAO), magnesium dechelatase (SGR), chlorophyllase (CLH), chlorophyll(ide) b reductase (NOL/NYC1), chlorophyllide a oxygenase (CAO) were measured by ELISA kits (JINGMEI, Jiangsu, China).

### Statistical analysis

Data was presented as the mean ± standard deviation (*n* = 3 or 10). The significant difference was conducted by independent-samples T test analysis at *p* < 0.05 and *p* < 0.01 by using SPSS 17.0 program (SPSS, Inc., Chicago, IL, U.S.A.). All the experiments were carried out three times except that the experiments of enzyme activity and photosynthetic parameter were carried out ten times. All figures were drawn using the software of GraphPad prism 6 (GraphPad Software, San Diego, CA, U.S.A.) and Adobe Photoshop CS5 (Adobe, San Jose, CA, U.S.A).

## Results

### Sensory evaluation

The sensory quality of field green tea (FT) and greenhouse green tea (GT) was compared in Table 1. Results showed that plastic greenhouse covering cultivation had a remarkable influence on appearance, aroma, liquor color, taste and total quality of green tea. The score of appearance, aroma, taste and total quality of FT was significantly higher than those of GT. Both aroma type and taste strength of FT was superior to GT. Nevertheless, GT had its own particular characteristics, such as greener appearance, liquor color, infused leaves and fresher taste. Collectively, the greenness of green tea could be remarkably improved by plastic greenhouse covering cultivation.

**Table 1:** Sensory evaluation of greenhouse green tea and field green tea

Samples	Appearance			Aroma			Liquor color			Taste			Infused leaf			Total score
	comment	score		comment	score		comment	score		comment	score		comment	score		
GT	slightly tight, bend, green, even	82.7 ± 1.5*		clean aroma	90.3 ± 0.6**		green, bright	87.7 ± 0.6*		refreshing	91.3 ± 1.2*		Soft, green, bright, slightly even	86.3 ± 1.5		88.1 ± 0.6**
FT	tight, bend, yellowish green, even	86.0 ± 1.0		bean-like aroma	94.0 ± 1.0		yellowish green, bright	84.3 ± 1.2		mellow, thick	94.3 ± 0.6		Soft, yellowish green, bright, even	88.3 ± 1.2		90.6 ± 0.2

*Note* Data are presented as mean ± standard deviation (*n* = 3); * indicates a significant difference at *P* < 0.05 level; ** indicates a significant difference at *P* < 0.01 level. GT, greenhouse tea; FT, field tea

### Color determination

In this study, the color difference value of both appearance and liquor of samples was analyzed (Table 2). From appearance aspect, the value of lightness difference (∆L) and red-green difference (∆a) of FT was significantly higher than that of GT, while the value of total color difference (∆E), hue (b/a), hue saturation (S_ab_) and hue angle (H_ab_) of GT was significantly higher that of FT. It suggested that the appearance color of GT was greener than FT, while the appearance of FT looked lighter than GT. From liquor aspect, the value of ∆L, the yellow-blue difference (∆b), b/a, hue chroma (C_ab_) and H_ab_ of GT was significantly higher than FT except that the ∆a value of GT was lower than FT, indicating that both liquor lightness and liquor greenness of GT was better than FT.

**Table 2:** Comparison of color difference value between field tea and greenhouse tea

Samples		∆L	∆a	∆b	∆E	b/a	C_ab_	S_ab_	H_ab_
Appearance	GT	-38.74 ± 1.55*	-9.70 ± 0.21**	24.28 ± 0.71	46.75 ± 0.95**	-2.81 ± 0.02**	29.97 ± 0.74	0.55 ± 0.01**	-1.23 ± 0.00**
	FT	-33.74 ± 1.57	-8.43 ± 0.22	24.00 ± 0.56	42.27 ± 1.02	-3.18 ± 0.03	29.30 ± 0.60	0.49 ± 0.01	-1.27 ± 0.00
Liquor	GT	-1.01 ± 0.12*	-2.62 ± 0.09**	13.30 ± 0.43*	13.59 ± 0.45	-4.13 ± 0.03*	15.06 ± 0.44*	0.49 ± 0.02	-1.33 ± 0.00*
	FT	-6.07 ± 1.25	-1.73 ± 0.09	11.93 ± 0.47	13.53 ± 0.90	-5.00 ± 0.33	13.54 ± 0.45	0.53 ± 0.04	-1.37 ± 0.01

*Note* Data are presented as mean ± standard deviation (*n* = 3); * indicates a significant difference at *P* < 0.05 level; ** indicates a significant difference at *P* < 0.01 level. GT, greenhouse tea; FT, field tea

### Chlorophyll determination

To further verify that GT was greener that FT, the chlorophyll content was determined as shown in Fig. 1. Figure 1B showed that the content of chlorophyll a and chlorophyll b of GT was significantly higher than those of FT. The total chlorophyll content of GT (4.15 mg∙g^− 1^) was about 1.6 times that of FT (2.54 mg∙g^− 1^). Similar trend was also observed in fresh tea leaves (Fig. 1C). The content of chlorophyll a, chlorophyll b and total chlorophyll in greenhouse fresh tea leaves (GFL) was significantly higher than those of field fresh tea leaves (FFL), and the total chlorophyll content of GFL (1.06 mg∙g^− 1^) was almost twice that of FFL (0.59 mg∙g^− 1^).

### Gene expression analysis

**Fig. 2:**
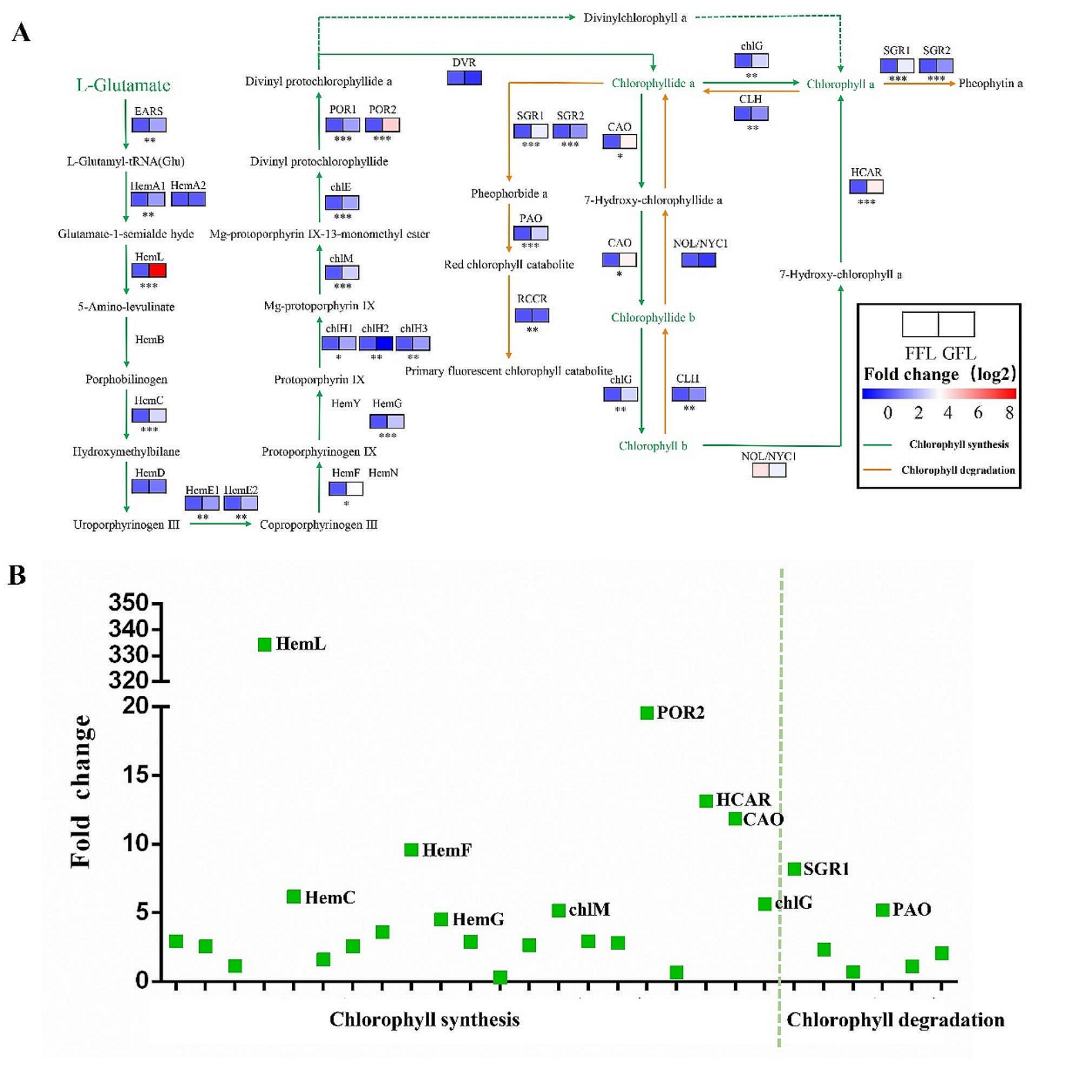
Effect of plastic greenhouse covering cultivation on the expression of genes related to chlorophyll metabolism. **A**, KEGG pathways of chlorophyll metabolism in tea leaves; **B**, fold change of relative expression of chlorophyll-metabolism-related genes under greenhouse covering cultivation. Data are presented as mean ± standard deviation (*n* = 3); * indicates a significant difference at *P* < 0.05 level; ** indicates a significant difference at *P* < 0.01 level; *** indicates a significant difference at *P* < 0.001 level. GFL, greenhouse fresh leaves; FFL, field fresh leaves

Chlorophyll in plants is formed by L-glutamic acid through a series of catalytic enzymes as shown in Fig. 2. A total of 30 genes were involved in chlorophyll metabolism in plants [23–25]. All these genes were quantitatively detected except *porphobilinogen synthase (HemB)*, *oxygen-independent coproporphyrinogen III oxidase (HemN)* and *protoporphyrinogen/coproporphyrinogen III oxidase (HemY)*, since they couldn’t be cloned in ‘Huangshanzhong’. The qRT-PCR results revealed that the expression of 27 genes in fresh tea leaves was strikingly affected by greenhouse covering cultivation, including 24 up-regulated genes and 3 down-regulated genes (Fig. 2B). L-glutamic acid can be directly transformed to L-glutamyl-tRNA by glutamyl-tRNA synthetase (EARS). The gene expression of *EARS* in GFL was about triple that of FFL (*p* < 0.01). 20 enzymes were found to participate in the conversion from L-glutamyl-tRNA to chlorophyllide a in tea plants. The gene expression of *glutamyl-tRNA reductase (HemA1)*, *glutamyl-1-semialdehyde-2,1-aminomutase (HemL)*, *hydroxymethylbilane synthase (HemC)*, *coproporphyrinogen synthase (HemE1/HemE2)*, *coproporphyrinogen III oxidase (HemF)*, *menaquinone-dependent protoporphyrinogen oxidase (HemG)*, *magnesium chelatase subunit (chlH1/chlH3)*, *magnesium-protoporphyrin O-methyltransferase (chlM)*, *magnesium-protoporphyrin IX monomethyl ester(oxidative) cyclase (chlE)* and *protochlorophyllide reductase (POR1/POR2)* showed similar trends with *EARS*, which was significantly higher in GFL than FFL. The gene expression of *glutamyl-tRNA reductase (HemA2)*, *uroporphyrinogen-III synthase (HemD)* and *divinyl chlorophyllide a 8-vinyl-reductase (DVR)* showed no noticeable variation between GFL and FFL.

Chlorophyllide a is a key synthetic precursor contributing to the formation of chlorophyll a and chlorophyll b. And all genes involved in 3 metabolic pathways of chlorophyllide a were also quantitatively detected. Firstly, chlorophyllide a can be directly synthesized to chlorophyll a by chlorophyll a synthase (chlG) and this reaction can be reversed by chlorophyllase (CLH). Our data revealed that, under greenhouse-covering cultivation, the expression of *chlG* and *CLH* in fresh tea leaves was significantly up-regulated by 5.6 times and 2.1 times respectively, indicating that the synthesis of chlorophyll a was intenser than the hydrolysis of chlorophyll a. Secondly, chlorophyllide a can be further transformed into chlorophyll b by chlorophyllide a oxygenase (CAO) and chlG. Chlorophyll b can be metabolized to chlorophyll a with reduction of chlorophyll(ide) b reductase (NOL/NYC1) and 7-hydroxymethyl chlorophyll a reductase (HCAR). *CAO* and *HCAR* gene expression was approximately 11- and 13-fold higher in GFL than in FFL respectively. Meantime, *CLH* and *NOL/NYC1* contribute to the conversion of chlorophyll b to chlorophyllide a, while unlike *CLH*, *NOL/NYC1* gene expression was not significantly different between GFL and FFL. Besides, with catalysis of magnesium dechelatase (SGR), pheophorbide a oxygenase (PAO) as well as red chlorophyll catabolite reductase (RCCR), chlorophyllide a can be further degraded into primary fluorescent chlorophyll catabolite. And the expression of these genes was significantly higher in GFL than in FFL.

### Enzyme activity analysis

The comparison of chlorophyll metabolism related enzymes activities between GFL and FFL was presented in Table 3. It was revealed that the enzyme activities of chlG, PAO, SGR and CAO in GFL were1.5-, 1.1-, 1.4-, and 1.2-fold higher than those in FFL respectively. While the enzyme activities of CLH and NOL/NYC1 in FFL were significantly higher than those in GFL. Our results suggested that the greenhouse-covering cultivation could improve chlorophyll contents primarily through increasing activities of chlorophyll synthesis related enzymes.

**Table 3:** Variation of enzyme activity related to chlorophyll metabolism between field green tea and greenhouse green tea (U/g)

	Chlorophyll a synthetase (chlG)	Pheophorbide a oxygenase (PAO)	Magnesium dechelatase (SGR)	Chlorophyllase (CLH)	Chlorophyll(ide) b reductase (NOL/NYC1)	Chlorophyllide a oxygenase (CAO)
GFL	0.86 ± 0.01**	3.40 ± 0.03**	26.85 ± 0.26**	2.08 ± 0.01***	0.27 ± 0.00**	24.94 ± 0.25**
FFL	0.56 ± 0.01	2.99 ± 0.04	18.64 ± 0.22	2.16 ± 0.02	0.38 ± 0.00	20.50 ± 0.26

*Note* Data are presented as mean ± standard deviation (*n* = 10); * indicates a significant difference at *P* < 0.05 level; ** indicates a significant difference at *P* < 0.01 level. GFL, greenhouse fresh leaves; FFL, field fresh leaves

### Photosynthetic parameter analysis

As indicated in Table 4, fresh tea leaves showed significant differences in intracellular CO_2_ (Ci), stomatal conductance (Gs), transpiration rate (Tr), water use efficiency (WUE) and net photosynthetic rate (Pn) after greenhouse covering. In comparison with FFL, Ci, Gs, Tr and Pn of GFL increased by 1.95 times, 2.81 times, 6.92 times and 1.59 times, respectively. A significant reduction of WUE was observed in fresh tea leaves under the greenhouse covering condition. For clarifying the correlation between chlorophyll content and photosynthetic parameters of fresh tea leaves, the Pearson correlation coefficient was analyzed (Table S2). Data revealed that chlorophyll content had a strong positive correlation with Pn, which suggested that the photosynthetic efficiency of greenhouse tea fresh leaves could be effectively improved by increasing chlorophyll content.

**Table 4:** Comparison of photosynthetic parameters between field tea leaves and greenhouse tea leaves

	Intracellular CO_2_ (Ci, µmol·mol^− 1^)	Stomatal conductance (Gs, mmol H_2_O·m^− 2^s^− 1^)	Transpiration rate (Tr, mmol H_2_O· m^− 2^s^− 1^)	Water use efficiency (WUE, mmol CO_2_· mol^− 1^H_2_O)	Net photosynthetic rate (Pn, µmol CO_2_· m^− 2^s^− 1^)
GFL	267.00 ± 21.80**	61.80 ± 14.78**	2.63 ± 0.42**	2.18 ± 0.62**	6.23 ± 2.86*
FFL	136.80 ± 75.30	22.00 ± 13.84	0.38 ± 0.21	6.50 ± 2.25	3.91 ± 1.48

*Note* Data are presented as mean ± standard deviation (*n* = 10); * indicates a significant difference at *P* < 0.05 level; ** indicates a significant difference at *P* < 0.01 level. GFL, greenhouse fresh leaves; FFL, field fresh leaves

## Discussion

The application of greenhouse has been popularized in agricultural field. With application of greenhouse, plants can grow and develop in a controllable and ideal condition. Moreover, the greenhouse facilitates plants to be grown in off-season, creating higher yield and more economic benefits [5]. Tea plants are prone to freezing and drought damage when growing in the northern tea areas of China. It has been proved that moderate stress can improve the quality of tea. For example, some abiotic stress (such as light, temperature, and mechanical damage) can enhance the expression levels of aroma synthetic genes, resulting in abundant accumulation of the characteristic aroma compounds in tea leaves [26, 27]. Moderate temperature difference between day and night can effectively improve the content of total free amino acid, caffeine and tea polyphenol of tea [28]. That is why the aroma and taste of FT are better than GT. Chlorophyll content is closely related to tea quality, which accounts for nearly 2/3 of all pigments in green tea [20, 29]. Our study found that the greenhouse-covering cultivation exhibited a similar effect with shading on increasing chlorophyll contents of tea leaves. A previous study found that the mono-layer cover of black net increased chlorophyll contents by 17.24%~25.00%, and the double-layer cover of black net increased more chlorophyll contents by 29.31%~40.63% [30]. However, shading led to thin leaves, shortened internodes as well as low photosynthetic rate, as a result, decreased tea yield [16]. Our results found that the plastic greenhouse cover increased chlorophyll contents by 63.38%~79.66%. Chlorophyll plays a vital role in absorption, transfer, and conversion of solar radiation. The net photosynthetic rate of covered leaves was 1.5 times higher than uncovered leaves. Therefore, our results suggested that the plastic greenhouse cover not only turned tea leaves green, but also caused a significant increase in tea yield. This change was primarily concerned with radiation intensity, temperature and relative humidity [31–33].

**Fig. 3:**
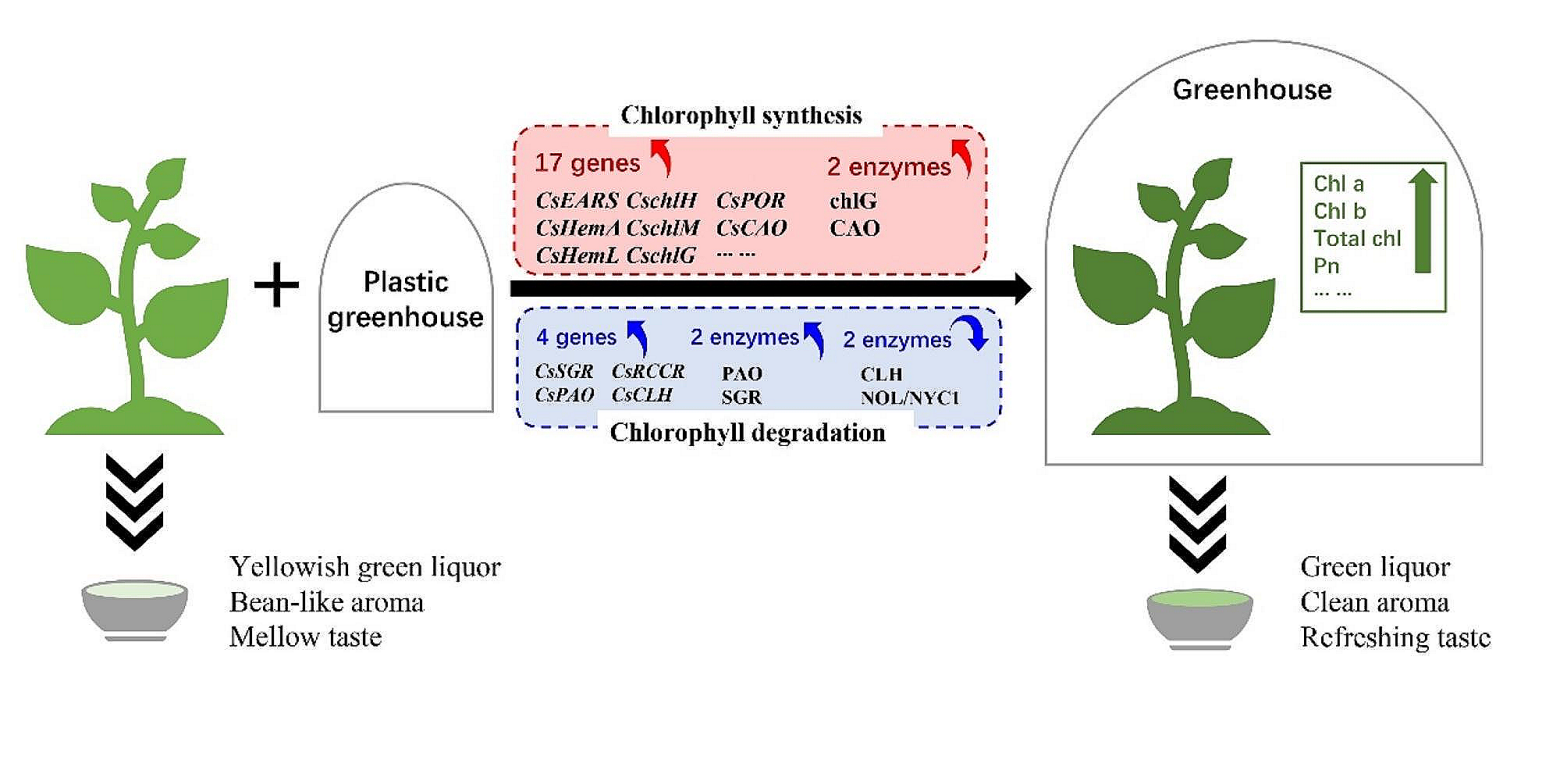
The effect of plastic greenhouse covering cultivation on chlorophyll in tea leaves

Chlorophyll is initially biosynthesized from L-glutamic acid, which is further converted to protochlorophyllide, triggering the ‘chlorophyll cycle’, which refers to the interconversion between chlorophyll a and chlorophyll b [34]. And the major enzymatic pathways and encoding genes associated with chlorophyll metabolism have been well illustrated in tea plant [12]. Accordingly, the gene expression and enzyme activity involved in chlorophyll metabolism in tea plant was also determined in this study. Totally, 23 genes were strikingly affected by greenhouse covering cultivation, among which 22 genes were significantly up-regulated. Recently, numerous studies have elucidated the relationship between environmental conditions and chlorophyll metabolism in plants. For instance, cold pretreatment could cause an acceleration of chlorophyll to degrade in green ripening bananas by activating *MaCBF1* to *MaCBR* and *MaSGR1* [35]. Postharvest heat treatments could delay chlorophyll degradation in Thai lime fruit [36]. Besides, drought stress significantly reduced the chlorophyll index (SPAD) of wheat [37]. The capability of chlorophyll fluorescence imaging of wheat decreased under frost and drought stresses [38]. It can be seen that low temperature and drought stress could prompt chlorophyll degradation. As known, the plastic greenhouse covering cultivation could protect tea plants against chilling damage and drought injury, which might be the chief cause that the greenhouse covered tea leaves had higher chlorophyll contents. Combined with the results of gene expression and enzyme activity, we considered that greenhouse covering cultivation could promote chlorophyll synthesis in tea fresh leaves by significantly increasing the gene expression and activities of chlorophyll metabolism related enzymes (Fig. 3). It has been proved that the Pn had a significantly positive correlation with the yield [39]. Our data showed Pn of greenhouse covered tea leaves was 1.5-fold higher than uncovered tea leaves, implying the yield of greenhouse tea was higher than field tea. However, further analysis of the differences in their quality composition is still needed.

## Conclusion

The color of greenhouse green tea was greener than field green tea. The chlorophyll content, intracellular CO_2_, stomatal conductance, transpiration rate, and net photosynthetic rate of greenhouse tea leaves was significantly higher than field tea leaves. Totally, 21 genes and 4 enzymes were significantly up-regulated by greenhouse covering cultivation, especially chlG, PAO and CAO. Therefore, the higher content of chlorophyll in greenhouse tea samples was primarily due to the higher expression of genes and activity of chlorophyll-metabolism-related enzymes in tea leaves covered by the greenhouse.

### Electronic supplementary material

Below is the link to the electronic supplementary material.

Supplementary Material 1

## Data Availability

All data generated or analyzed during this study are included in this published article.

## Abbreviations

FT: Field tea
GT: Greenhouse tea
FFL: Field fresh leaves
GFL: Greenhouse fresh leaves
EARS: Glutamyl-tRNA synthetase
HemA: Glutamyl-tRNA reductase
HemL: Glutamate-1-semialdehyde-2,1-aminomutase
HemB: porphobilinogen synthase
HemC: Hydroxymethylbilane synthase
HemD: Uroporphyrinogen-III synthase
HemE: Coproporphyrinogen synthase
HemF: Coproporphyrinogen III oxidase
HemN: Oxygen-independent coproporphyrinogen III oxidase
HemY: Protoporphyrinogen/coproporphyrinogen III oxidase
HemG: Menaquinone-dependent protoporphyrinogen oxidase
chlH: Magnesium chelatase subunit H
chlM: Magnesium-protoporphyrin O-methyltransferase
chlE: Magnesium-protoporphyrin IX monomethyl ester(oxidative) cyclase
POR: Protochlorophyllide reductase
DVR: Divinyl chlorophyllide a 8-vinyl-reductase
SGR: Magnesium dechelatase
PAO: Pheophorbide a oxygenase
CAO: Chlorophyllide a oxygenase
chlG: Chlorophyll a synthase
NOL/NYC1: Chlorophyll(ide) b reductase
CLH: Chlorophyllase
HCAR: 7-hydroxymethyl chlorophyll a reductase
RCCR: Red chlorophyll catabolite reductase
