# Supplementary material for: Greenhouse covering cultivation promotes chlorophyll accumulation of tea plant (Camellia sinensis) by activating relevant gene expression and enzyme activity
Source: BMC Plant Biol. 2024 May 24;24:455. doi: 10.1186/s12870-024-05149-7 (PMC11127325; doi:10.1186/s12870-024-05149-7)
Supplement: Supplementary file 1 — Supplementary Material 1 [file 12870_2024_5149_MOESM1_ESM.pdf]

Table S1 Chlorophyll-metabolism-related genes real-time fluorescent quantitative PCR primer.

Gene Name	Primer for qRT-PCR(5'-3')
CsEARS-Q-F	CTCCTGGTTCTCCTCCTA
CsEARS-Q-R	GCATACTCTAGCCATTCATC
CsHemA1-Q-F	ATTCGTTGCGAGATTGTT
CsHemA1-Q-R	GCTGCTCCTTTCCTTTGT
CsHemA2-Q-F	GTTCAAGACCGCAATCACCG
CsHemA2-Q-R	TGCCTGCTCCAACAACCAA
CsHemL-Q-F	GGGAACTTGGGCTTCATCG
CsHemL-Q-R	CACTTAGAGTGTCGGCTTGGTAC
CsHemC-Q-F	TGACCGCCATTCTTTCTA
CsHemC-Q-R	GCTAATCTTGTTTCCTCGT
CsHemD-Q-F	TGTCTGGGCTGTCTTCGA
CsHemD-Q-R	CAAATCAGGCAACCGTGT
CsHemE1-Q-F	AGTGGATATGGCAGATGG
CsHemE1-Q-R	CAGATTGAGAATGTGACCC
CsHemE2-Q-F	ACATTCGCTTCTGTTCCC
CsHemE2-Q-R	TTTCTACTTCCAGCCCTC
CsHemF-Q-F	GACTTCACGCATCATCTTC
CsHemF-Q-R	ATTTGAATCGGAATCGTC
CsHemG-Q-F	TCTGTGGAAGAAACGGAACT
CsHemG-Q-R	CCGCAACGAAAGGGTCAA
CschlH1-Q-F	AGGTCTCAGTTCCGTGTT
CschlH1-Q-R	AAGGCATAGTTTCATCTCA
CschlH2-Q-F	CCCATCTGGTTGCTTCGT
CschlH2-Q-R	TTGGCTGTTCCTCGCTTT
CschlH3-Q-F	TGCTGCTTACTACTCGTTT
CschlH3-Q-R	CGCTCATTCCCACCTGCT
CschlM-Q-F	CTCTATTGCCTCATTCCTC
CschlM-Q-R	ATTTAGTGTTTGGGTTGGT
CschlE-Q-F	ATTTAGTGTTTGGGTTGGT
CschlE-Q-R	ATTCTTTGGTGTTGAGGC
CsPOR1-Q-F	CTACGACAGGCTTGTTTAGGGA
CsPOR1-Q-R	CTGGTTTTCAAATGAGGCAGAA
CsPOR2-Q-F	GTGATGTGGAGAAAGCTCGTAAA
CsPOR2-Q-R	CTAGAAGAAAACAAGGCGACTGA

Gene Name	Primer for qRT-PCR(5’-3’)
CsDVR-Q-F	CAAACCTATCACAGCCTCAACC
CsDVR-Q-R	CCAACACCACTCCTTTCCCT
CsSGR1-Q-F	ATGGGTACTTTGACTGCT
CsSGR1-Q-R	TTGGGTGATCTTCTTCTG
CsSGR2-Q-F	CACGGACCTACACCCTCT
CsSGR2-Q-R	GCCCGCTCACATAACAAT
CsNYC1-Q-F	GGGAGGTAGCCGAGAAAG
CsNYC1-Q-R	AAAGCACCCAAAGCATCA
CsHCAR-Q-F	TCCAGACCCATTCCTCCT
CsHCAR-Q-R	AATCTTCTTTCCTCCCTC
CsPAO-Q-F	TGGCAATAACCAACCTGA
CsPAO-Q-R	CAAATGAGCCGATTAAGAA
CsRCCR-Q-F	GTGGCTATTCCTTCTTGG
CsRCCR-Q-R	CAGTGCTCCTGCTGGTAA
CsCAO-Q-F	ACCTTGGGTTATCTTTCGTGG
CsCAO-Q-R	AGGAATGGTGGCTGTTGGA
CsCLH-Q-F	TAGGCAAAGTAACAACTCCATCC
CsCLH-Q-R	CGAGCCAATCACCAAAACC
CschlG-Q-F	GGTATCAAAGGAGCCAAGCAA
CschlG-Q-R	GTAAGACAGGGGCCAGACATTA
CsGADPH-Q-F	TTGGCATCGTTGAGGGTCT
CsGADPH-Q-R	CAGTGGGAACACGGAAAGC

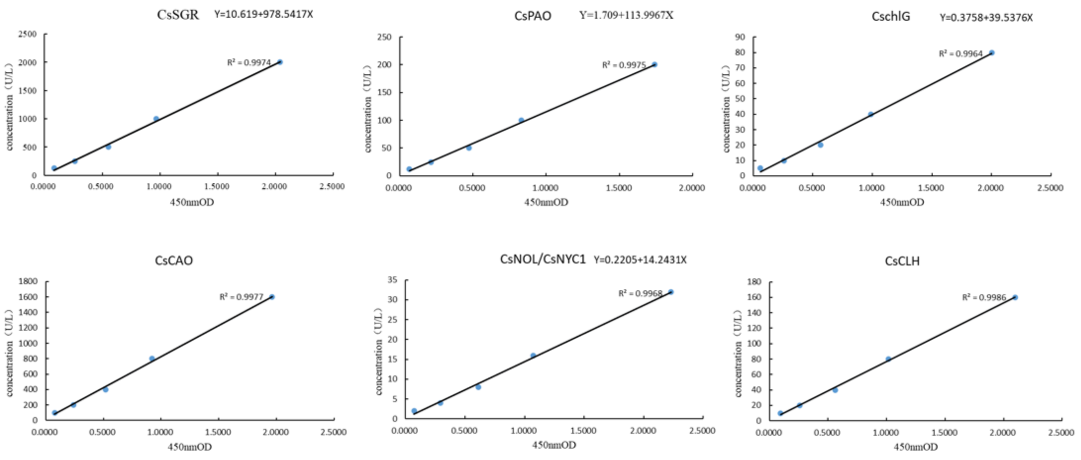

Figure S1 Standard curve of chlorophyll-metabolism-related enzyme activity

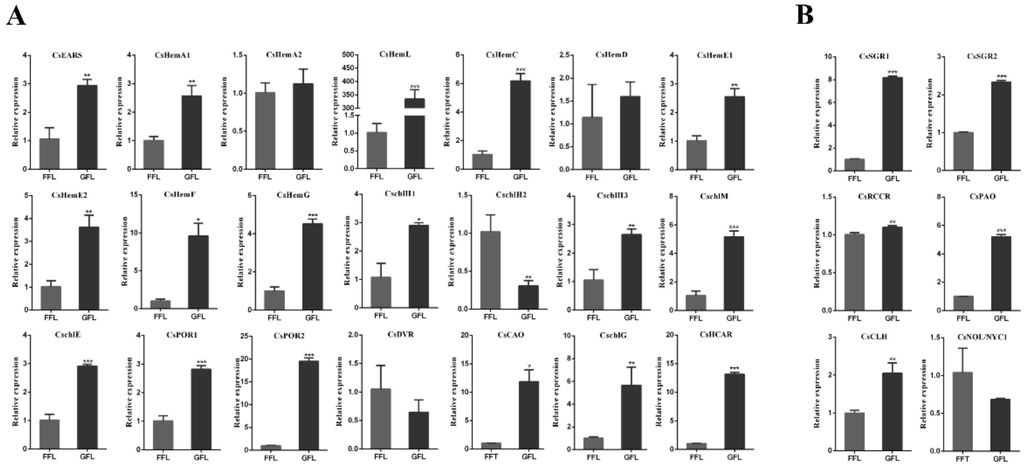

Figure S2 Effect of plastic greenhouse covering cultivation on the expression of genes related to chlorophyll metabolism. A, relative expression level of chlorophyll synthesis genes by qRT-PCR using CsGAPDH as the housekeeping gene, B, relative expression level of chlorophyll degradation genes by qRT-PCR using CsGAPDH as the housekeeping gene. data are presented as mean ± standard deviation (n = 3); * indicates a significant difference at $p < 0.05$ level; ** indicates a significant difference at $p < 0.01$ level; *** indicates a significant difference at $p < 0.001$ level. GFL, greenhouse fresh leaves; FFL, field fresh leaves.

Table S2 Pearson analysis of chlorophyll content and photosynthetic parameters

		Intracellular CO_2 (Ci, $\mu L \cdot L^{-1}$)	Stomatal conductance (Gs, mol $H_2O \cdot m^{-2}s^{-1}$)	Transpiration rate (Tr, $mmol \cdot m^{-2}s^{-1}$)	Water use efficiency (WUE, $g \cdot kg^{-1}$)	Net photosynthetic rate (Pn)
chlorophyll contents	1st leaf	0.462*	0.494*	0.587**	-0.401	0.705**
	2nd leaf	0.482*	0.589**	0.673**	-0.502*	0.602**

Note: * indicates a significant difference at $p < 0.05$ level; ** indicates a significant difference at $p < 0.01$ level.

Table S3 The environmental factors of FT and GT

**Sample**	**Date**	**Temperature**	**Relative humidity**	**Carbon dioxide concentration**
FT	2023.4.28 Sunny	10~32℃	32~44%	500~1200 ppm
	2023.4.29 Cloudy	11~29℃	33~48%	498~1000 ppm
	2023.4.30 Cloudy	12~29℃	37~51%	560~998 ppm
	2023.5.1 Sunny	12~30℃	34~47%	552~1002 ppm
	2023.5.2 Rainy	11~26℃	32~52%	487~980 ppm
GT	2023.4.28 Sunny	16~42℃	50~62%	632~1084 ppm
	2023.4.29 Cloudy	15~40℃	47~59%	589~1132 ppm
	2023.4.30 Cloudy	16~43℃	47~64%	552~1098 ppm
	2023.5.1 Sunny	17~44℃	42~62%	660~1123 ppm
	2023.5.2 Rainy	15~39℃	55~66%	603~1043 ppm

Note: GT, greenhouse tea; FT, field tea.
